# Epigenomics: dissecting hybridization and polyploidization

**DOI:** 10.1186/s13059-017-1254-7

**Published:** 2017-06-19

**Authors:** Scott A. Jackson

**Affiliations:** Center for Applied Genetic Technologies, University of Georgia, Riverbend Drive, Athens, GA 30602 USA

## Abstract

Epigenetic profiling in diploid, allopolyploid, and domesticated cotton shows that despite most DNA methylation being conserved and stably inherited, alterations likely due to hybridization and domestication affect gene expression.

## Investigating polyploidization in a cotton model

Polyploids are prevalent among plants and polyploidy is considered to contribute to genetic and phenotypic novelty. For example, many crop plants that have exaggerated organ (fruit) sizes are polyploid. Polyploidy has long fascinated biologists. Ohno [1] proposed that gene duplication through polyploidy was probably important in the evolution of species and of genetic complexity. The genetic complexities of “combining” independent genomes (allopolyploidy) into a common nucleus has long perplexed scientists.

**Fig. 1:**
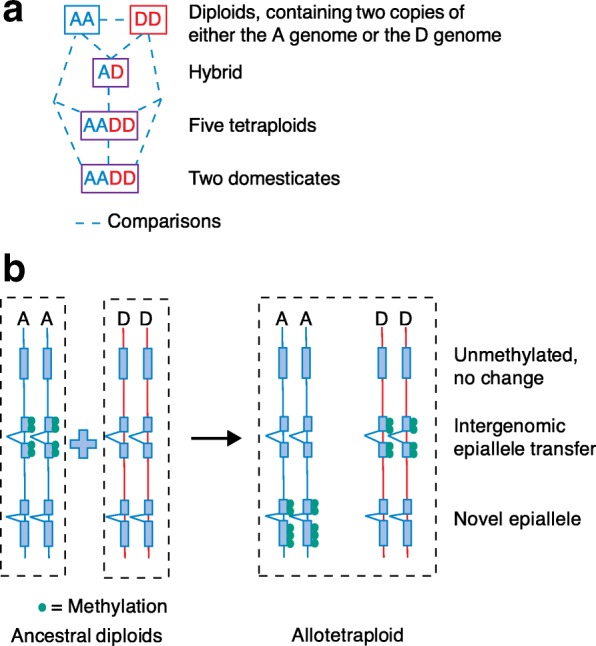
Schema of the comparisons made among the cotton species and various ploidies. **a** AA and DD = ancestral diploid genome types. AD = diploid hybrid. AADD = allotetraploid. *Dotted line* shows comparisons made in Song et al. [2]. **b** Genes shown as *blue boxes* on *vertical lines* for AA and DD diploids and an AADD tetraploid. *Green circles* illustrate DNA methylation showing from *top* to *bottom*: no methylation in diploids and no methylation in allotetraploid; transfer of methylation from the A genome of a diploid to the D subgenome in an allotetraploid; and finally, the formation of a novel epiallele that is not observed in the two ancestral diploids

In a recent publication in *Genome Biology*, Song et al. [2] used the cotton genus, *Gossypium*, to explore the consequences of polyploidization and subsequent domestication on the epigenome, specifically the methylome. The elegant system that they used involved representatives of two ancestral diploids (referred to as AA and DD), a synthetic hybrid (AD), five allotetraploids (AADD) that formed around 1–1.5 million years ago, and domesticated forms derived from two of the wild allotetraploids (AADD) (Fig. 1a). This system allowed the authors to explore epigenetic changes that resulted from hybridization and/or polyploidy, as well as changes that are correlated with much more recent domestication events.

## Most DNA methylation is conserved over millions of years

Not surprisingly, and concordant with other recent publications [3], Song et al. [2] found that cytosine methylation (mC) was, for the most part, highly heritable and stable—even when millions of years separate the diploid genomes from their counterpart subgenomes in the polyploids. There were surprisingly few mC epiallelic differences among the accessions, though epiallelic diversity was more frequent than nucleotide variation. Moreover, epiallelic changes corresponded with an increased frequency of transposable elements (TEs) in the host genome, which suggests a role for TEs in maintaining heritable epiallelic diversity. Additionally, TEs are interlinked with de novo epiallele formation and this may be a source of variation that should be further explored in the context of adaptation and domestication.

It has been shown that genome-wide mC levels are often associated with TE content [4]; thus, it was not surprising that the A genome, which is twice the size of the D genome, had higher levels of mC because of its increased TE content. Despite the higher level of overall methylation in the A genome, the D genome had higher levels of gene body mCG, probably because of the presence of more intergenic and methylated TEs in the D genome. This was only true for mCG and mCHG, as mCHH levels were similar between the A and D genomes. One conclusion is that, while having higher levels of TEs and mC, the A genome was more efficient than the D genome in compartmentalizing these elements and epigenetic marks other than mC into the pericentromeric regions; thus, the A genome has lower levels of gene-body methylation.

An unanswered question in the study of allopolyploids is the role of the wide hybridization of two divergent genomes into a common nucleus versus that of the subsequent polyploidy of the two genomes. Song et al. [2] found that a majority of nearly 70,000 hybrid-induced epialleles were conserved in the five allopolyploid species. The process of hybridization in cotton, then, appears to contribute to the de novo formation of epialleles, which are then inherited stably in the derived polyploids. This also indicates that the formation of epialleles can be recapitulated in subsequent hybridizations of the parental species, as the polyploids are millions of years old but the A × D hybrid was made only recently.

## Epigenomic changes in polyploids

The additional copies of genes in a polyploid provide genetic information that can be acted on through mutation and selection to provide novel genotypes and phenotypes. Interestingly, the authors found that, when compared to the wild diploids, the allotetraploids had hundreds of thousands of CG and CHG differentially methylated regions (DMRs) that were enriched in genic and intergenic regions. These DMRs can affect the evolutionary trajectory of genes. Their role in the allopolyploids in establishing or contributing to the asymmetrical evolution of subgenomes remains to be determined. In cotton [5], as in other species [6], it has been observed that one subgenome is dominant over the other because it has higher gene expression levels and lower levels of gene loss. To what extent, if any, have the DMRs affected the differential gene loss, gene expression, or evolution of the subgenomes?

Song et al. [2] found many changes in the CG methylation of genes that were correlated with changes in gene expression. A small percentage of mCG DMRs were flipped between the A and D genomes when occurring in the tetraploid species *G. hirsutum*; that is, if a locus was mCG in the A genome of one ancestral diploid and not methylated in the D genome of the other, in *G. hirsutum*, the locus was mCG in the D genome and unmethylated in the A genome (Fig. 1b). Moreover, these genes were more likely to have divergent expression patterns in the tetraploid. This raises the question of how the methylation patterns changed and whether it was concomitant to homoeolog expression divergence, or alternatively, a result of this process?

Gene loss is common in polyploids (e.g., *Arabidopsis* [7]), especially for those that have undergone fractionation. Here, Song et al. [2] examined genes in the ancestral diploids and found that mC genes were more likely to be lost in the resultant polyploids, suggesting that the mC mark precedes either hybridization or polyploidization. This finding should be tempered, however, as these genes are also commonly enriched for TEs, mis-annotated as genes, may have been previously duplicated by polyploidy or other duplication processes, and may be already undergoing loss in the diploids. In any case, these data show that information that promotes the loss of mC genes is maintained during hybridization and polyploidy.

## A role for epigenetic reprogramming in domestication

The extent to which epigenetic variation contributes to either domestication or subsequent breeding is largely unknown. In the case of cotton, two allotetraploids, *G. hirsutum* and *G. barbadense*, were domesticated independently*.* Although a majority of mCG DMRs are not shared between the two domesticates, Song et al. [2] found a small set of 519 genes that are associated with shared DMRs. This is a candidate set of domesticated epialleles that may have been independently selected on during both domestications. Among these 519 genes are two clear examples of reprogrammed epialleles that putatively affect domestication traits.

One example of a shared domestication epiallele was *COLD2d,* a homolog of *Arabidopsis CONSTANS* (*CO*), a photoperiod-sensitive gene that regulates flowering. *COL2D* genes were found to have lower levels of CG methylation and higher levels of expression in both cultivated forms of cotton than in their undomesticated antecedents. Both domesticated cotton species are photoperiod insensitive, a major part of the domestication syndrome. Moreover, analysis of several accessions of wild and domesticated cotton species showed a clear correlation between decreased mCG levels in *COL2D* and increased expression of the gene.

## Remaining questions

The power of the system used here to explore evolutionary aspects of the epigenetic landscape lies in the number of intra-generic comparisons: two wild diploids could be compared to a hybrid, several derived allopolyploids, and two independently domesticated cottons. Despite the power of the experimental system, several questions remain to be addressed in future studies. First, how representative is cotton? The results in cotton contrast with those of work in *Arabidopsis* in that the initial hybrid had generally lower levels of DNA methylation [8]. Are there general rules for how methylation is regulated in hybrids, or is it species- or even cross-specific?

In the context of the polyploids, how is DNA methylation shared or transferred between the subgenomes, particularly at the level of genes? Song et al. [2] observed “transfer” of methylation between homoeologous loci, with resulting changes in gene expression. It may be that this is regulated through some small RNA pathway, or there could be a physical association between subgenomes that could lead to inter-genomic transfer of DNA methylation. How often does this occur and is it dependent on similarity between chromosomes (homology)? Does it contribute to variation within constrained populations, such as breeding programs?

Similarly, how important is gene methylation for either dosage balance or sub-/neo-/non-functionalization of paralogs in polyploids? Song et al. [2] observed that genes that are methylated in non-CG contexts in the diploids were more likely to be lost in the polyploids, but they did not address possible subfunctionalization of paralogs, changes in expression patterns across tissues, or gene-dosage compensation where stoichiometric balances need to be maintained.

The example of the *COL2D* epiallele and its contribution to the loss of photoperiod sensitivity is intriguing, but we do not know how often epialleic variants have contributed to domestication traits. Other cloned domestication genes in other species have not been epialleles, and in fact most have been transcription factors (reviewed by Meyer and Purugannan [9]). Are we missing epiallelic variants, or are they rare? As epiallelic variation accumulates more rapidly than variation in single-nucleotide polymorphisms (SNPs), to what extent does it contribute to adaptation or even response to selection in plant improvement?

Finally, there is a limitation in how these findings can be extended within this genus. Song et al. [2] observed that about 30% of DMRs were conserved among the five polyploids and that non-mCHH DMRs were enriched in genic and intergenic regions. These mCG and mCGH epialleles could certainly contribute to diversification through the regulation of genes. The limitation here is that only a single accession was sampled for each polyploid. What is required is a pan-epigenome approach that will allow us to truly understand epiallelic variation within a species at a more granular level. This will enable us to have a better grasp on what is conserved versus what is more recently derived.

## Conclusions

Like other reports, Song et al. [2] show that most DNA methylation is transmitted vertically and faithfully, and that there are few de novo changes in DNA methylation. In the context of polyploidy, they show that what changes they do find in DNA methylation occur primarily through hybridization and not via polyploidy per se. Finally, they do show that epiallelic variation may have contributed to the domestication of cotton.

## Abbreviations

cM: Cytosine methylation
DMR: Differentially methylated region
TE: Transposable element
